# Supplementary material for: A versatile platform for sequential glyco-, phospho-, and proteomics with multi-PTMs integration
Source: Nat Commun. 2026 Jan 28;17:948. doi: 10.1038/s41467-025-68270-7 (PMC12852849; doi:10.1038/s41467-025-68270-7)
Supplement: Supplementary file 1 — Supplementary Information [file 41467_2025_68270_MOESM1_ESM.pdf]

Supplementary Information

Xuefang Dong[1,2,7], Fangfang Xiong[1,2,7], Guangzhu Du[1,3], Yunfei Yang[4,5], Cheng Chen[1,2], Yun Cui[2], Xinlian Ding[1,6], Xiuling Li[1,2*], Yidong Shen[4,5*], Xinmiao Liang[1,2*]

[1]State Key Laboratory of Phytochemistry and Natural Medicines, Dalian Institute of Chemical Physics, Chinese Academy of Sciences, Dalian 116023, P. R. China

[2]Ganjiang Chinese Medicine Innovation Center, Nanchang 330100, P. R. China

[3]Department of Materials Science and Engineering, Dalian Maritime University, Dalian 116026, P. R. China

[4]Department of Geriatrics, The First Affiliated Hospital of Chongqing Medical University, Chongqing 400016, P. R. China

[5]State Key Laboratory of Cell Biology, Shanghai Institute of Biochemistry and Cell Biology, Center for Excellence in Molecular Cell Science, Chinese Academy of Sciences, Shanghai 200031, P. R. China.

[6]University of Chinese Academy of Sciences, Beijing 100049, PR China

[7]These authors contributed equally.

*Corresponding author. Email: lixiuling@dicp.ac.cn (X. L.); yidong.shen@sibcb.ac.cn (Y. S.); liangxm@dicp.ac.cn (X. L.);

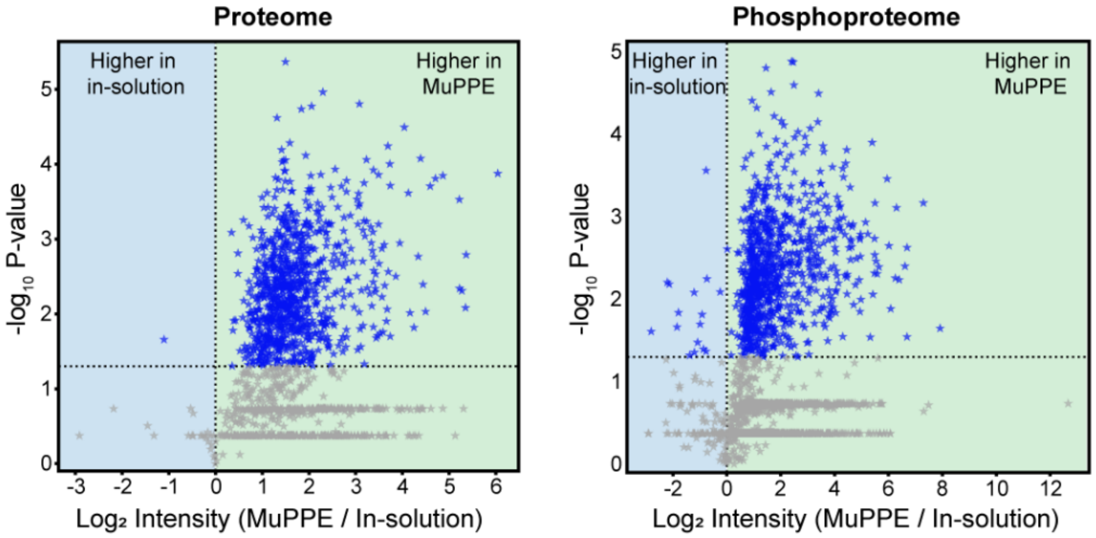

Supplementary **Fig. 1.** Paired TMT comparison of MuPPE vs in-solution workflows (n = 3). Volcano plots for the global proteome (left) and phosphoproteome (right). Each point represents a protein group (left) or a phosphopeptide (right). The x-axis shows within-run $\log_2$ intensity ratios (MuPPE/In-solution) computed after isotopic correction; the y-axis shows $-\log_{10}$ P from paired t-tests across three matched 2-plex TMT runs. Blue stars indicate features significantly higher in MuPPE at the stated thresholds; grey points are not significant. Under equal input and TMT multiplexing, MuPPE yields higher and more consistent quantitative signal than the in-solution workflow across both the proteome and phosphoproteome layers.

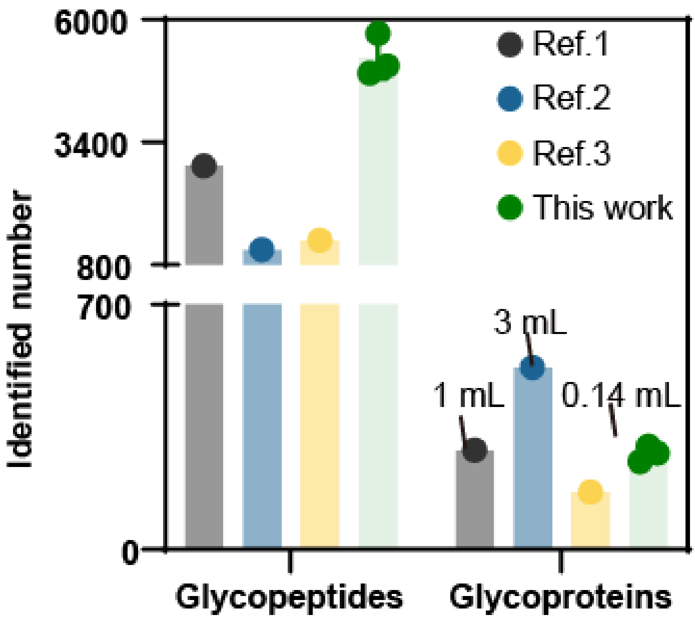

Supplementary **Fig. 2.** Comparison of reported N-linked glycopeptides and N-linked glycoproteins of HCSF in previous work and this study[1, 2, 3].

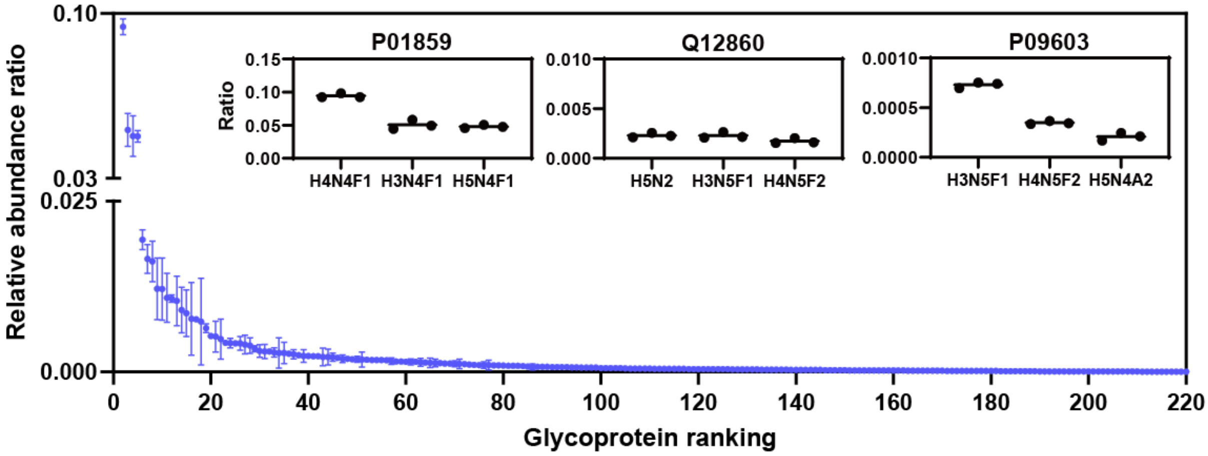

Supplementary **Fig. 3.** The reliable glycosylation quantification of HCSF. Distribution of coefficient of variance in glycoproteins across triplicates with overlap of identified glycopeptides in MuPPE.–The coefficient of variation distribution across different glycoproteins for N-linked glycan type quantification (Inset) also demonstrated the robustness of MuPPE (n = 3).

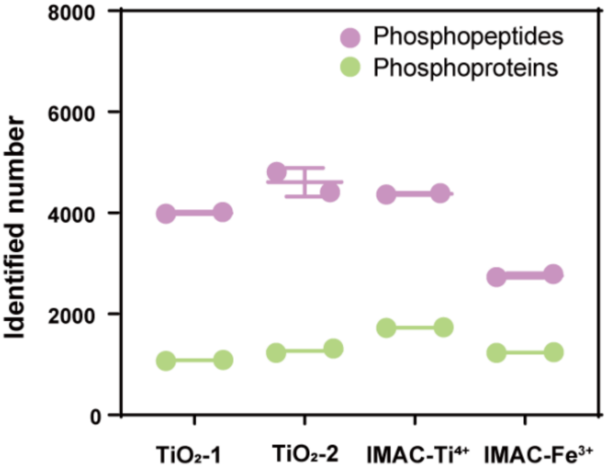

Supplementary **Fig. 4.** The identification results of phosphopeptides and phosphoproteins from different operators or using different materials with MB lysates ("TiO_2-1" and "TiO_2-2" correspond to experiments executed by operator-1 and operator-2, respectively, n = 2).

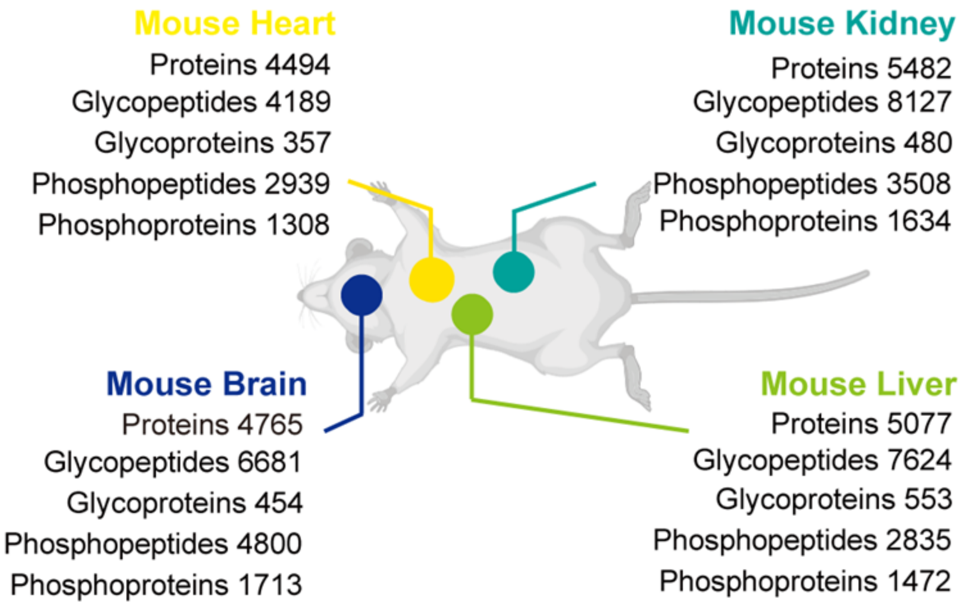

Supplementary **Fig. 5.** Proteomic, glycoproteomic, and phosphoproteomic analyses of four mouse tissues with MuPPE (n = 2). Created in BioRender. Dong. (2025) [https://BioRender.com/2qv24ya]

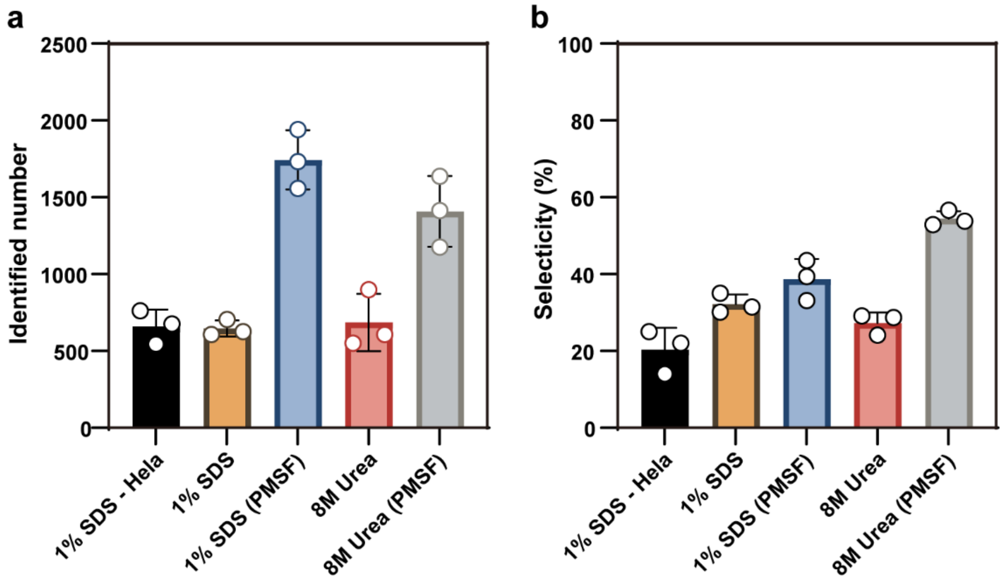

Supplementary **Fig. 6.** Investigation of effect of distinct lysis buffer on phosphopeptides enrichment (n = 3). **a.** the identified number of phosphopeptides. **b.** the selectivity of enriched phosphopeptides. We infer that this phenomenon, which aligns with observations reported in other studies, demonstrated this protein aggregation instability likely due to the charge properties of the phosphopeptide enrichment materials, adversely affected the subsequent enrichment results.

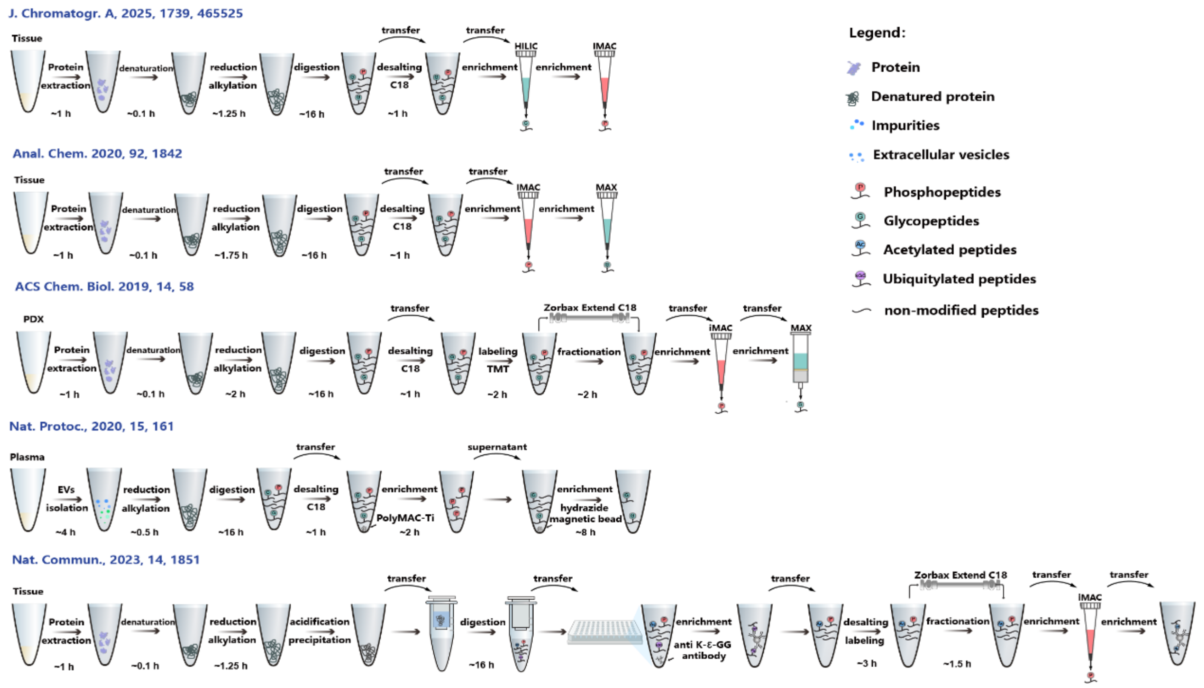

Supplementary **Fig. 7.** Summary diagram of the sequential enrichment process for different glycopeptides and phosphopeptides within state-of-the-art platforms according to the reports.

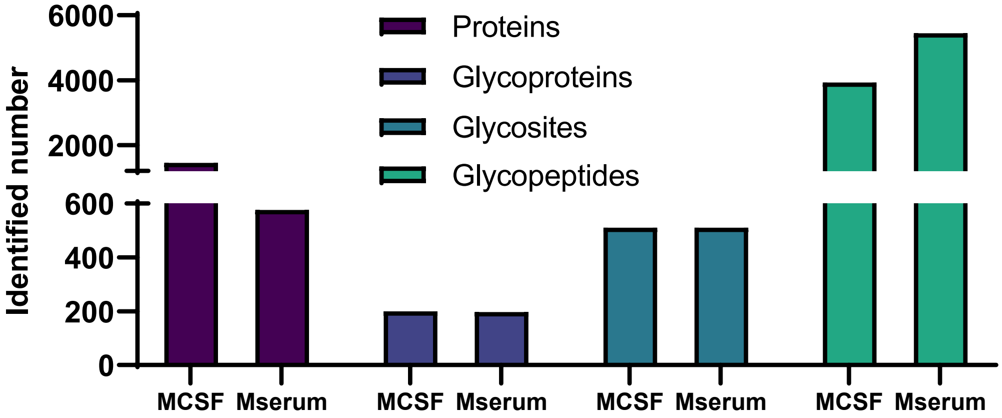

Supplementary **Fig. 8.** Overview of the number of identified proteins, glycoproteins, glycosites, and glycopeptides in mice cerebrospinal filed (MCSF) and mice serum (Mserum) samples (n = 6).

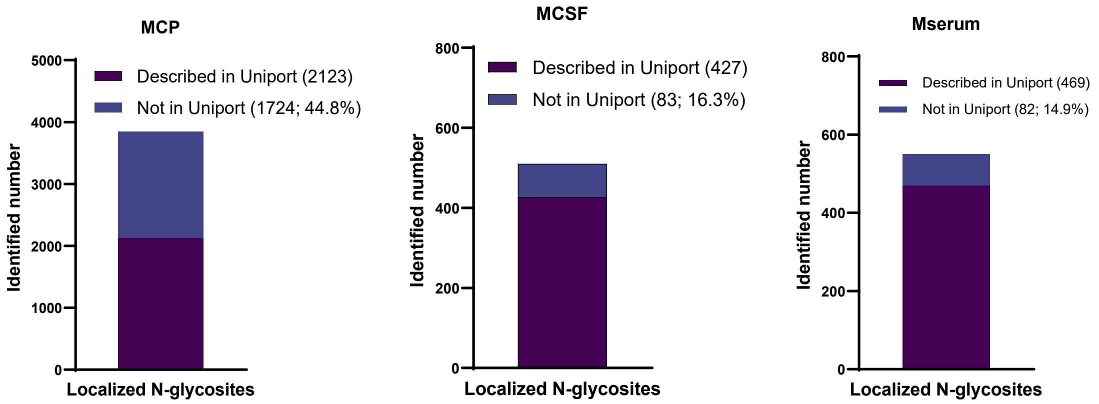

Supplementary **Fig. 9.** The novelty of glycosites identified in Mserum, MCSF, and mice choroid plexus (MCP) samples relative to those reported in UniProt, as summarized in the GlyGen dataset (Version 2.6.1, released on 08/07/2024).

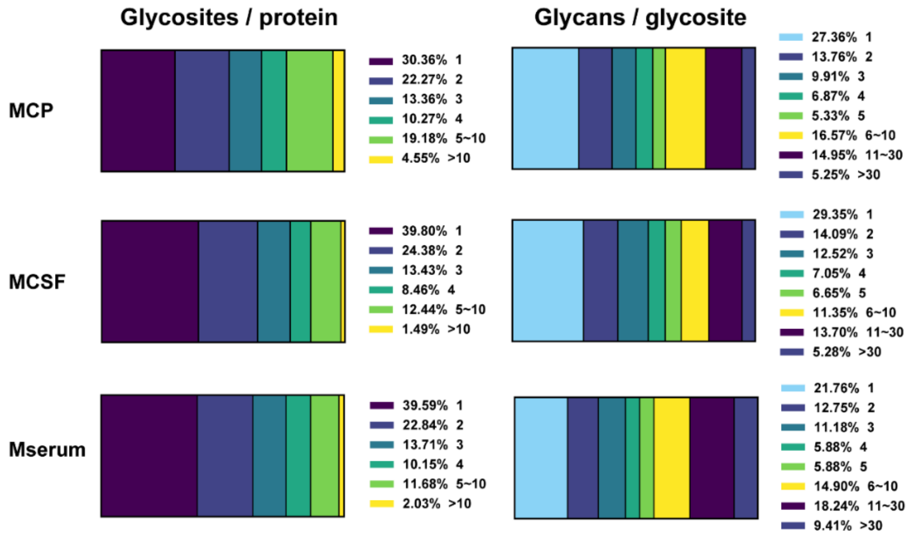

Supplementary **Fig. 10.** Distribution of glycosite numbers per glycoprotein (left) and the diversity of glycans at each glycosite (right) in MCP, MCSF and Mserum samples, illustrating both macro- and micro-heterogeneity of glycoproteins.

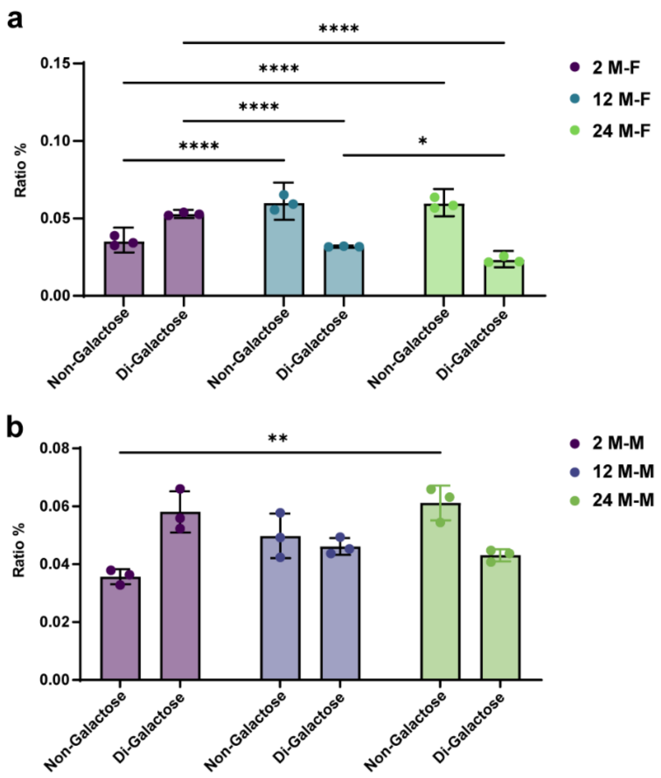

Supplementary **Fig. 11.** Relative abundance of galactosylation in females (a) and males (b) at different age groups in MCP samples, comparison by ANOVA followed by Tukey's multiple comparisons test ($P < 0.05$) (n = 3). Galactosylation levels are higher in females than in males, with a marked decline in females during midlife, whereas males exhibit a gradual decrease over time.

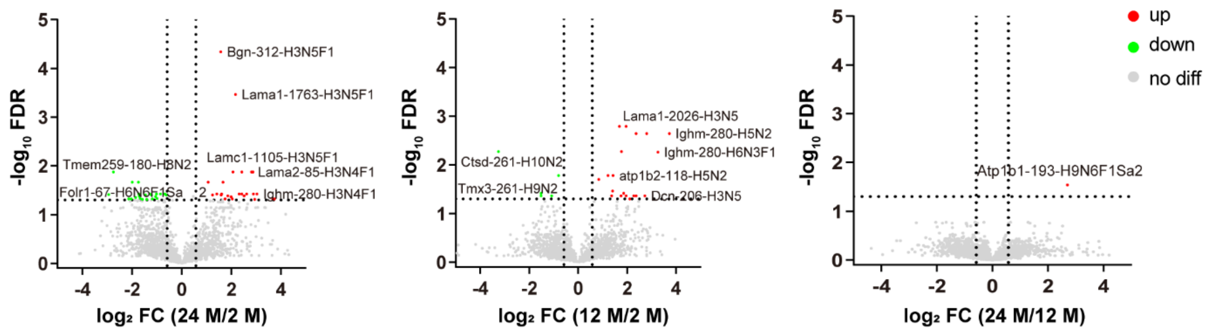

Supplementary **Fig. 12.** Volcano plots show the fold change (FC) and -log$_{10}$ FDR (y-axis) for the overall association of pairwise glycopeptides in 2 M, 12 M, and 24 M in MCP samples (n = 6) (FDR<0.05; FC>1.5).

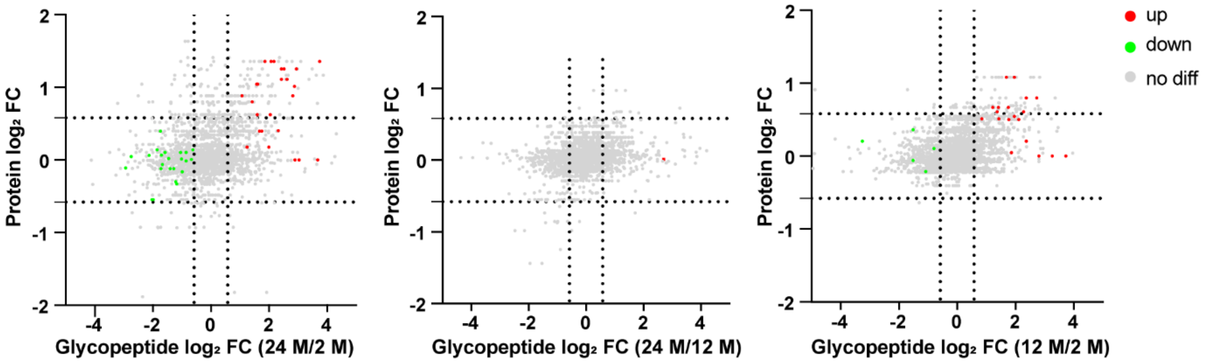

Supplementary **Fig. 13.** Median glycosites FC compared to the protein FC in each compared group in MCP samples (n = 6). It shows that PTM changes are not driven by protein-level shifts in our data. All primary PTM analyses therefore use peptide-level quantification, with matched protein values reported in Supplementary Data.

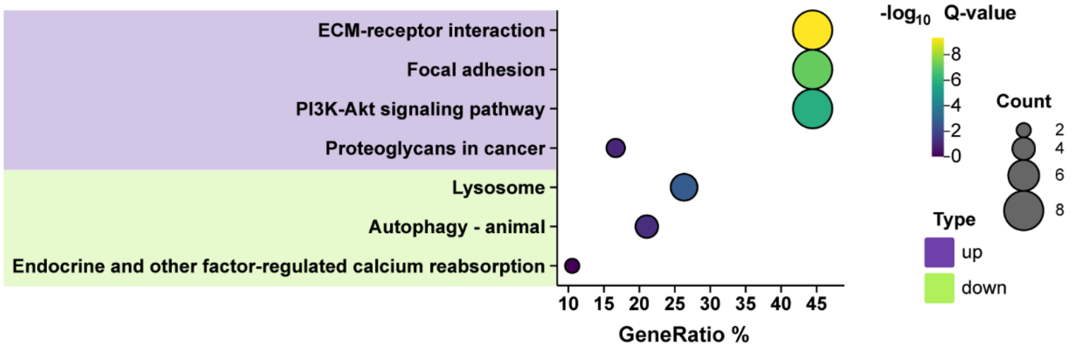

Supplementary **Fig. 14.** Kyoto Encyclopedia of Genes and Genomes (KEGG) pathway enrichment analysis was performed on the glycoproteins corresponding to the upregulated (purple) and downregulated (green) glycopeptides in the MCP samples of 24 M/2 M mice. Circle size indicates glycopeptide count, and color represents $-\log_{10}$ Q-value.

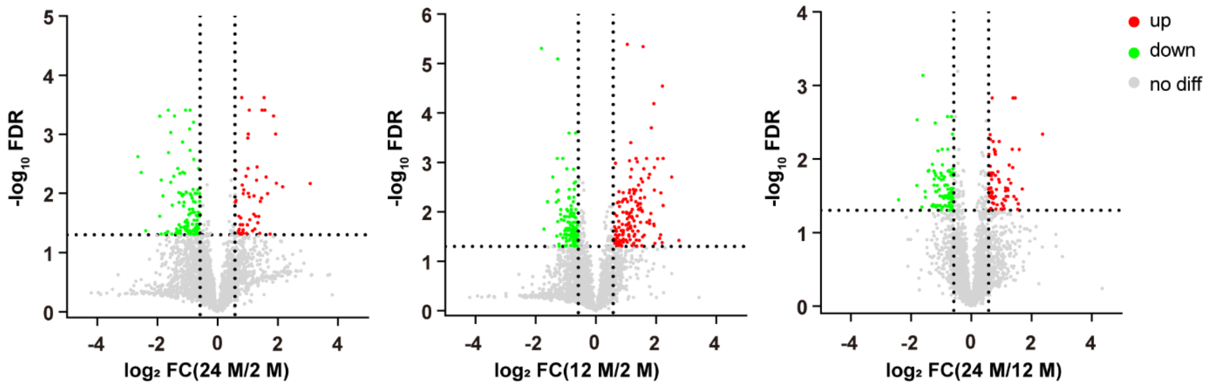

Supplementary **Fig. 15.** Volcano plots show the FC and -$\log_{10}$ FDR (y-axis) for the overall association of pairwise phosphosites in 2 M, 24 M, and 24 M in MCP samples (n = 6) (FDR<0.05; FC>1.5).

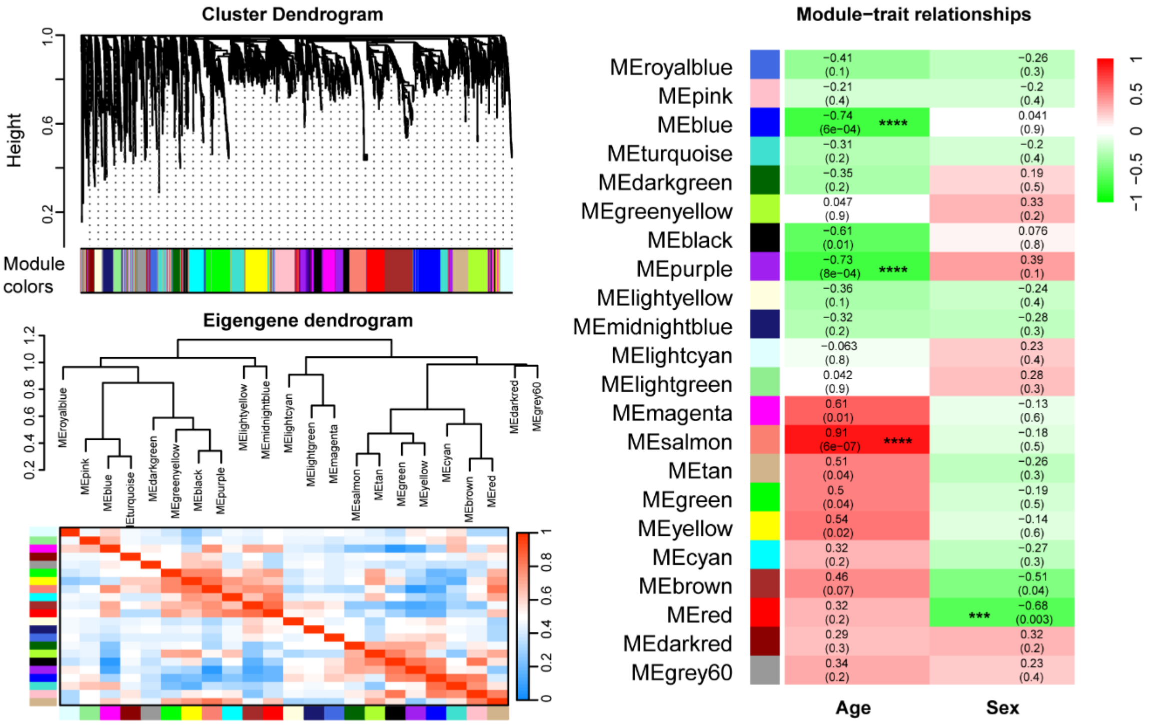

Supplementary **Fig. 16.** Phosphosite co-regulation network analysis identified three age-related modules and one sex-related module in MCP samples.

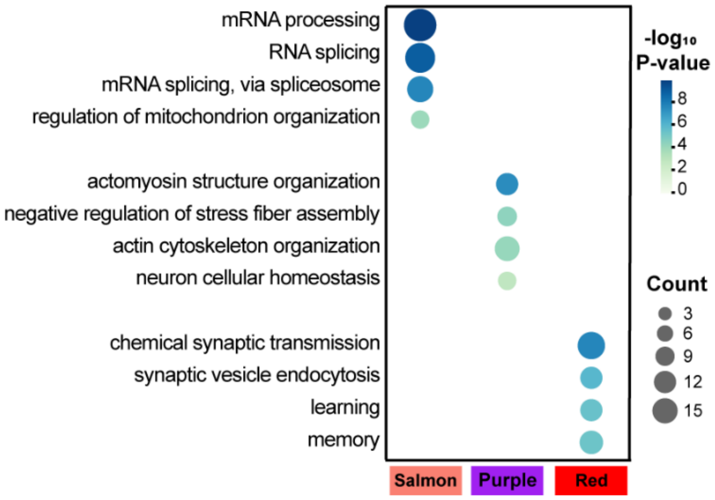

Supplementary **Fig. 17.** GO analysis of biological process categories enriched in each aging-associated module shown with Benjamini-Hochberg FDR-corrected *P* values. Count indicates the number of phosphoproteins per GO term. The results showed that the modules positively correlated with age were enriched in the functions related to mRNA, RNA processing and splicing, while the negatively correlated modules were most enriched in actomyosin structure organization, negative regulation of stress fiber assembly and neuronal cell homeostasis.

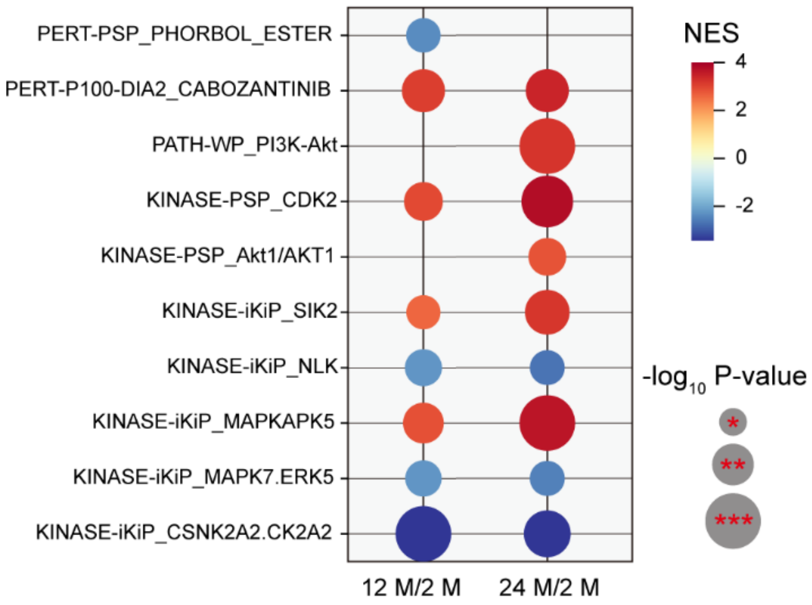

Supplementary **Fig. 18.** PTM signature enrichment analysis (PTM-SEA) bubble plot for 12 M/2 M and 24 M/2 M contrasts. Circles are coloured by normalized enrichment scores (NES) (red, positive enrichment; blue, negative) and scaled by significance (bubble area, proportional to $-\log_{10}$ P-value); signature names are shown on the left.

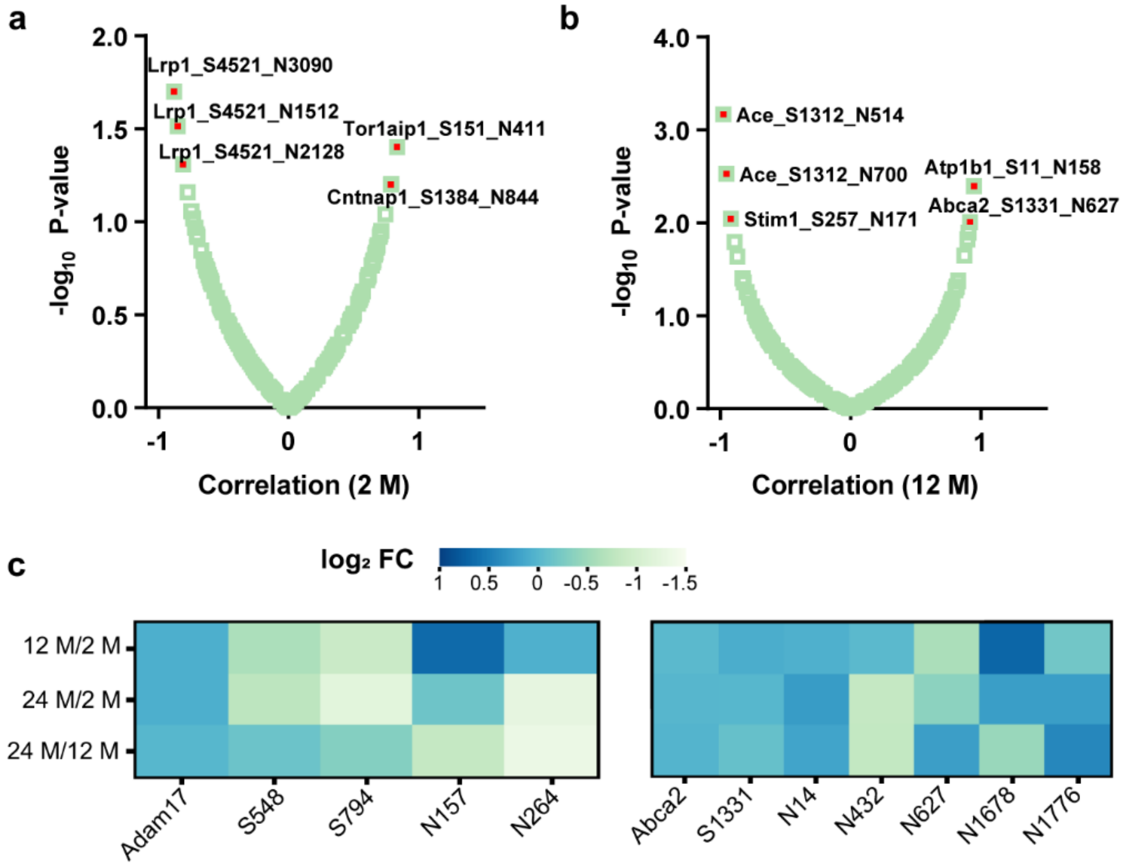

Supplementary **Fig. 19.** Glycosylation/phosphorylation site correlation in 2 M (a) and 12 M (b) group, with scatterplots showing correlation coefficients (x-axis) and -log$_{10}$ P-values (y-axis); **c** Heatmaps display log$_2$ FC of representative glycosylated and phosphorylated protein forms (e.g., *Adam17, Abca2*) across age groups (2 M, 12 M, and 24 M), highlighting dynamic age-dependent post-translational modifications in MCP samples (n = 6).

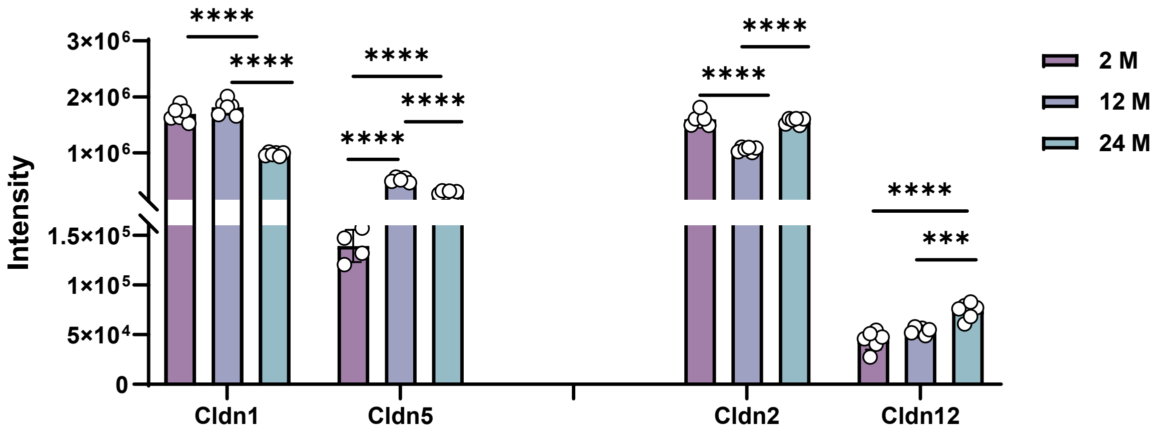

Supplementary **Fig. 20.** Changes in the expression levels of Cldn protein family in MCP samples at different age groups, comparison by ANOVA followed by Tukey's multiple comparisons test ($P < 0.05$) (n = 6).

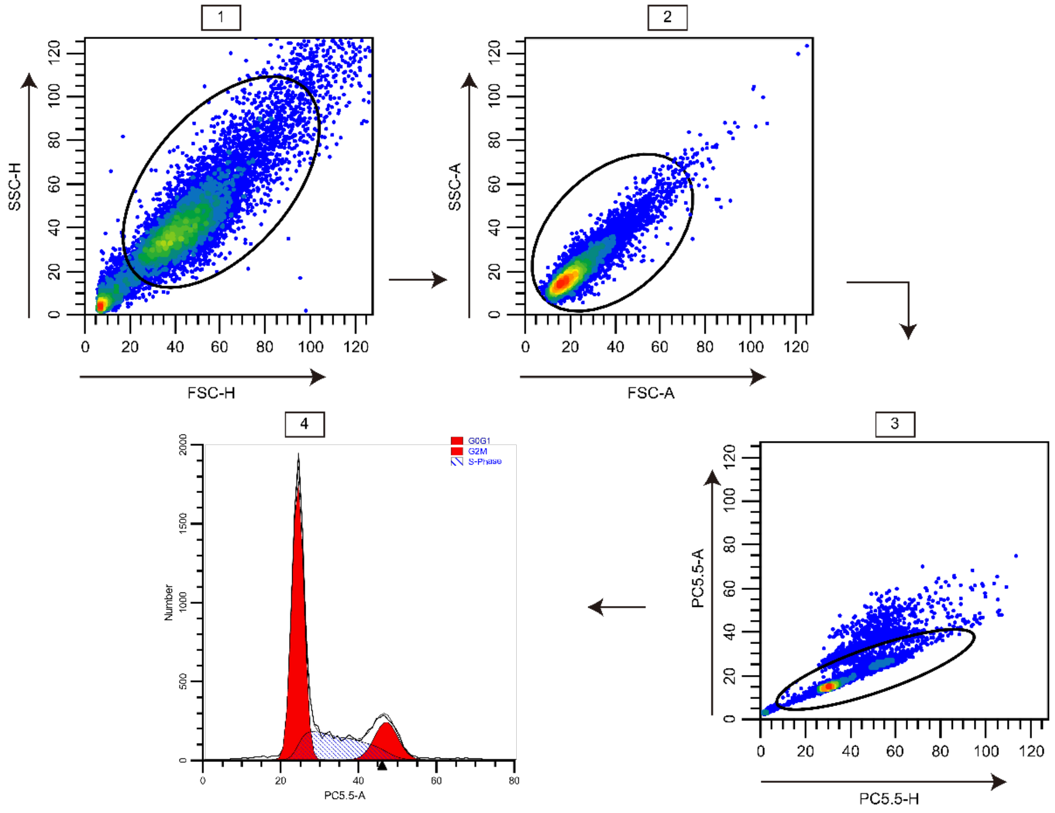

Supplementary **Fig. 21.** The schematic gating strategy for Fig. 6j.

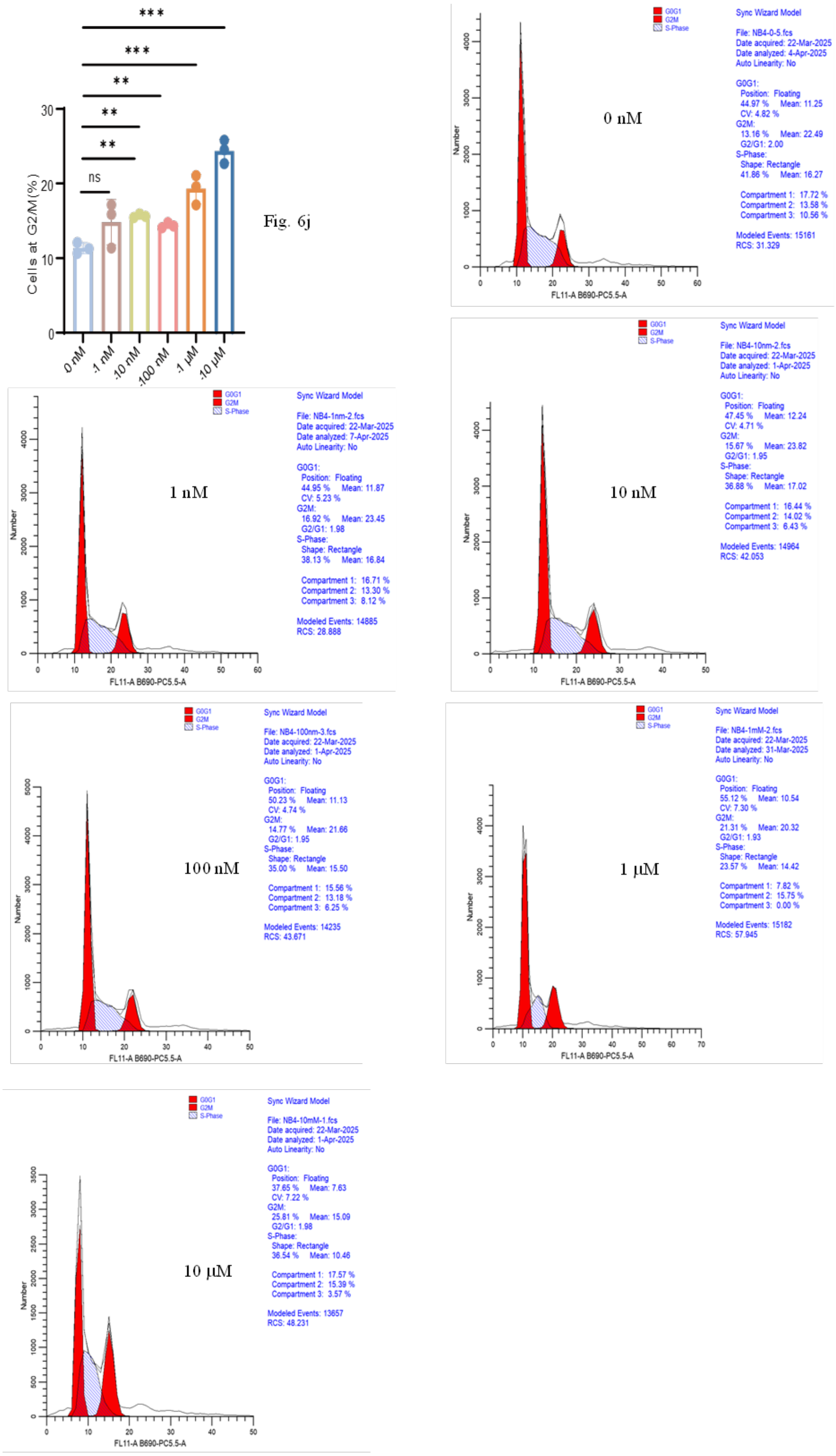

Supplementary **Fig. 22.** The cross-referenced panels correspond to which data panels in **Fig. 6j** (n = 3).

Appendix. The relevant parameters for the referenced studies for Supplementary **Fig. 2.**

	Title	Journal	Year	Removal of high abundant protein	CSF Volume /mL	Enrichment Materials	No. of glycopep	No. of glycopro.
Ref. 1	In-depth Site-specific Analysis of N-glycoproteome in Human Cerebrospinal Fluid and Glycosylation Landscape Changes in Alzheimer's Disease	Molecular & Cellular Proteomics	2021	No	1	HILIC and boronic acid enrichment	2893	285
Ref. 2	In-depth Characterization of the Cerebrospinal Fluid (CSF) Proteome Displayed Through the CSF Proteome Resource (CSF-PR)	Molecular & Cellular Proteomics	2014	Yes	3	magnetic hydrazide beads	1121	520
Ref. 3	Boost-DiLeu Enhanced Isobaric N, N-Dimethyl Leucine Tagging Strategy for Comprehensive Quantitative Glycoproteomic Analysis	Analytical Chemistry	2022	No	not mentioned	SAX-HILIC	1321	165
This work				No	0.142	Click-Mal	5018	251

Methods.

Mice sample collection: To collect brain, heart, liver, and kidney samples, mice were anesthetized with an intraperitoneal injection of Avertin (20 mg/ml) and then euthanized with a lethal dose to ensure humane treatment. Transcardial perfusion with ice-cold phosphate buffered saline (PBS) was performed for 5 min to remove circulating blood and immune cells. After perfusion, the skull was carefully opened, and the brain was dissected, rinsed in ice-cold PBS to remove residual blood, and flash-frozen in liquid nitrogen. The thoracic and abdominal cavities were then opened to expose the heart, liver, and kidneys. These organs were carefully excised using surgical scissors, rinsed with ice-cold PBS to remove excess blood, and promptly flash-frozen in liquid nitrogen. During subsequent processing, protease and phosphatase inhibitor cocktails were applied to preserve protein integrity for downstream MuPPE analysis, and all samples were stored at -80 °C.

To collect CSF, serum, and CP for MuPPE analysis, 14 female and 14 male mice were included in each age group. CSF collection was adapted from a previous report[4]. Briefly, mice were anesthetized intraperitoneally with Avertin (20 mg/ml), and then placed in a stereotactic instrument with the head secured at a 45-degree angle facing downwards. An incision was made above the neck and muscles were held separated with microretractors allowing exposure of the cisterna magna by blunt forceps without any bleeding. CSF was collected from the cisterna magna cavity with a glass capillary connected to a hydraulic microinjection instrument. CSF was centrifuged at 1,000 x g for 5 min at 4 °C to exclude blood cell contamination. Supernatant was collected and treated with protease and phosphorylase inhibitor cocktail and kept at -80 °C.

To collect serum, the whole blood of the mice post anesthesia was collected through enucleation of the eyeball. Blood was left to sit at room temperature for 30 min, followed by a 3, 000 x g centrifugation at 4°C for 10 min. The supernatant was further centrifuged at 4°C and 10,000 x g for 10 min. Serum was collected and treated with protease and phosphorylase inhibitor cocktail and kept at -80 °C.

To collect CP, mice were administered a lethal dose of Avertin, then transcardially perfused with ice-cold PBS for 5 min to remove any circulating immune leukocytes from the central nervous system vasculature. Following perfusion, brains were rapidly extracted and placed in ice-cold PBS. CPs from lateral ventricles (LVs) and fourth ventricle (4V) were rapidly dissected under a stereomicroscope. CPs from 14 mice in each group were pooled together, immediately snap-frozen in liquid nitrogen and stored at -80°C.

Beads information: Click-Maltose (Click-Mal), Click-Cys and HBS beads employed were both home-made according to our previous work [5, 6, 7]. The commercial ZIC-HILIC beads were obtained from Merck's SeQuant® ZIC®-HILIC HPLC columns. Amino-NH_2 bonded beads and Strong-anion exchange (SAX) beads were obtained from Zhejiang Acchrom Technology Co., Ltd.

Glycopeptides enrichment with Click-Mal: Adjust the tryptic sample solution to 80% acetonitrile (ACN)/1% trifluoroacetic acid (TFA) by adding ACN and TFA and incubate for 10 min for glycopeptides enrichment. Centrifuge the mixture, retain the Click-Mal beads and transfer the supernatant to a new Eppendorf (EP) tube for further phosphopeptides enrichment and proteomic analysis. The Click-Mal beads were washed with 40 μL 80% ACN/1% TFA, and then transferred to the tip and washed twice again with 30 μL 80% ACN/1% TFA to remove non-glycosylated peptides. The glycopeptides were eluted with 30% ACN/1% formic acid (FA) twice, dried down, and reconstituted in 0.1% FA prior to LC-MS/MS analysis[5].

Glycopeptides enrichment with Click-Cys: Adjust the tryptic sample solution to 80% ACN/2% FA by adding ACN and FA and incubate for 10 min for glycopeptides enrichment. Centrifuge the mixture, retain the Click-Cys beads. The Click-Cys beads were washed with 50 μL 80% ACN/2% FA, and then transferred to the tip and washed twice again with 50 μL 80% ACN/2% FA to remove non-glycosylated peptides. The glycopeptides were eluted with 50 μL 0.1% FA twice, dried down, and reconstituted in 0.1% FA prior to LC-MS/MS analysis[6].

Glycopeptides enrichment with HBS: Adjust the tryptic sample solution to 80% ACN/2% FA by adding ACN and FA and incubate for 10 min for glycopeptides enrichment. Centrifuge the mixture, retain the HBS beads. The HBS beads were washed with 50 μL 80% ACN/2% FA, and then transferred to the tip and washed twice again with 50 μL 80% ACN/2% FA to remove non-glycosylated peptides. The glycopeptides were eluted with 0.1% FA twice, dried down, and reconstituted in 0.1% FA prior to LC-MS/MS analysis[7].

Glycopeptides enrichment with ZIC-HILIC: Adjust the tryptic sample solution to 80% ACN/0.2% TFA by adding ACN and trifluoroacetic acid and incubate for 10 min for glycopeptides enrichment. Centrifuge the mixture, retain the ZIC-HILIC beads. The ZIC-HILIC beads were washed with 50 μL 80% ACN/0.2% TFA, and then transferred to the tip and washed twice again with 50 μL 80% ACN/0.2% TFA to remove non-glycosylated peptides. The glycopeptides were eluted with 40 μL 30%ACN/2% FA twice, dried down, and reconstituted in 0.1% FA prior to LC-MS/MS analysis[8].

Glycopeptides enrichment with Amino-NH_2: Adjust the tryptic sample solution to 83% ACN/1% FA by adding ACN and FA and incubate for 10 min for glycopeptides enrichment. Centrifuge the mixture, retain the Amino-NH_2 beads. The Amino-NH_2 beads were washed with 40 µL 83% ACN/1% FA, and then transferred to the tip and washed twice again with 40 µL 80% ACN/1% FA and 40 µL 70% ACN/1% FA to remove non-glycosylated peptides. The glycopeptides were eluted with 50 µL 40% ACN/5% FA twice, dried down, and reconstituted in 0.1% FA prior to LC-MS/MS analysis.

Glycopeptides enrichment with SAX: Adjust the tryptic sample solution to 70% ACN/0.1% acetic acid/10 mM ammonium acetate by adding ACN and acetic acid and incubate for 10 min for glycopeptides enrichment. Centrifuge the mixture, retain the SAX beads. The SAX beads were washed with 40 µL 70% ACN/0.1% acetic acid/10 mM ammonium acetate, and then transferred to the tip and washed twice again with 40 µL 70% ACN/0.1% acetic acid/10 mM ammonium acetate and 40 µL 60% ACN/0.1% acetic acid/10 mM ammonium acetate to remove non-glycosylated peptides. The glycopeptides were eluted with 50 µL 50% ACN/0.1% acetic acid/10 mM ammonium acetate twice, dried down, and reconstituted in 0.1% FA prior to LC-MS/MS analysis[9].

Phosphopeptides enrichment with commercial IMAC-Ti^{4+}: Adjust the flow-through condition for phosphopeptide enrichment according to the manufacturer’s instructions, and then incubate for 10 min to facilitate phosphopeptide enrichment. Centrifuge the mixture, retain the IMAC-Ti^{4+} beads and transfer the supernatant for proteome identification. IMAC beads were sequentially washed with 50 µL 80% ACN/6% TFA, 2 × 100 µL 50% ACN/6% TFA/200 mM NaCl, 2 × 200 µL 30% ACN/0.1% FA, and 2 × 100 µL 30% ACN. Phosphopeptides were finally eluted twice with 30 µL 10% ammonia solution and pooled.

Phosphopeptides enrichment with TiO_2: Adjust the flow-through condition for phosphopeptide enrichment according to the manufacturer’s instructions, and then incubate for 10 min to facilitate phosphopeptide enrichment. Subsequently, transfer the sample to the extraction tip and wash sequentially with 80% ACN/ 5% TFA/1 M glycolic acid, followed by 80% ACN/1% FA, and lastly with 20% ACN/0.1% FA. Elute the phosphopeptides using 30 µL of 10% ammonia solution twice, then concentrate the eluate by evaporation and reconstitute it in 0.1% FA in preparation for LC-MS/MS analysis.

LC-MS/MS analysis of different proteomic samples

Samples were loaded onto capillary analytical column packed in-house with C18 1.9 mm ReproSil particles (Dr. Maisch GmbH), with an EASY-nLC 1200 system (Thermo Fisher Scientific) coupled to the MS (Orbitrap 480, Thermo Fisher Scientific). The column oven maintained column temperature at 50°C. The mobile phases consisted of 0.1% FA (A) and 0.1% FA and 80% ACN (B).

For the proteomic samples, peptides were separated through a gradient of up to 90% buffer B over 20 min at a flow rate of 1 μL/min. The gradient of the mobile phase started from 3% B to 15% B for 0.5 min and then was increased linearly to 50% B in 15 min, to 90% in 1.5 min, and maintained for 3 min. The LC-MS/MS system was operated in data-independent MS/MS acquisition mode. The full mass scan acquired in the Orbitrap mass analyzer was from *m/z* 350 to 1500 with a resolution of 60000 (*m/z* 200). The MS/MS scans were also acquired by using an Orbitrap with a 30000 resolution (*m/z* 200), and the AGC target was set as custom. The spray voltage and the temperature of the ion transfer capillary were set to 2.6 KV and 320 °C, respectively. The normalized collision energy for HCD and dynamic exclusion was set as 32% and 30 s, respectively.

For the glycoproteomic samples, peptides were separated through a gradient of up to 90% buffer B over 120 min at a flow rate of 600 nL/min. The gradient of the mobile phase started from 3% B to 10% B for 6 min and then was increased linearly to 40% B in 10 min, to 90% in 4 min, and maintained for 10 min. The LC-MS/MS system was operated in data-dependent MS/MS acquisition mode. The full mass scan acquired in the Orbitrap mass analyzer was from *m/z* 350 to 1500 with a resolution of 60000 (*m/z* 200). The MS/MS scans were also acquired by using an Orbitrap with a 30000 resolution (*m/z* 200), and the AGC target was set as standard. The spray voltage and the temperature of the ion transfer capillary were set to 2.6 KV and 320 °C, respectively. The normalized collision energy for HCD and dynamic exclusion was set as 20%, 30%, 40% and 30 s, respectively.

For the phosphoproteomic samples, peptides were separated through a gradient of up to 90% buffer B over 60 min at a flow rate of 600 nL/min. The gradient of the mobile phase started from 1% B to 5% B for 3 min and then was increased linearly to 32% B in 50 min, to 90% in 2 min, and maintained for 5 min. The LC-MS/MS system was operated in data-independent MS/MS acquisition mode. The full mass scan acquired in the Orbitrap mass analyzer was from *m/z* 350 to 1500 with a resolution of 60000 (*m/z* 200). The MS/MS scans were also acquired by using an Orbitrap with a 30000 resolution (*m/z* 200), and the AGC target was set as custom. The spray voltage and the temperature of the ion transfer capillary were set to 2.6 KV and 320 °C, respectively. The normalized collision energy for HCD and

dynamic exclusion was set as 32% and 30 s, respectively.

For TMT labeling proteomic and phosphoproteomic samples: peptides were separated through a gradient of up to 90% buffer B over 90 min at a flow rate of 600 nL/min. The gradient of the mobile phase started from 2% to 5% B for 1 min and then was increased linearly to 40% B in 70 min, to 45% in 10 min, to 100% in 5 min, and maintained for 5 min. The LC-MS/MS system was operated in data-dependent MS/MS acquisition mode. MS1 scans (350-1500 *m/z*) were acquired at a resolution of 60,000 with an AGC target of 200 % and a maximum injection time of 5 ms. MS/MS scans were acquired with 0.7 *m/z* isolation windows (scan range 110-3000 *m/z*), 59 windows, a maximum injection time of 100 ms and an AGC target of 300 %. The spray voltage and the temperature of the ion transfer capillary were set to 2.6 KV and 320 °C, respectively. The normalized collision energy for HCD and dynamic exclusion was set as 34% and 30 s, respectively.

TMT labeling experimental process: For the MuPPE-TMT workflow, approximately 20 μg of mouse brain proteins (lysed in 8 M urea/HEPES) were reduced with 1.1 μL of 200 mM DTT (final concentration 10 mM, 37 °C, 60 min) and alkylated with 1.12 μL of 800 mM IAA (final concentration 40 mM, dark, 30 min). Each sample was incubated with 0.6 mg maltose beads (5 μL suspension in water) and 63 μL ACN (final concentration 70%) for 10 min, centrifuged at 4000 x g for 4 min, and washed three times with 200 μL of 80% ethanol. Beads were resolubilized in 8 μL of 6 M urea, sonicated to disperse, diluted with 40 μL of 100 mM TEAB, and digested with 2 μL trypsin (0.5 μg, 1:40) at 37 °C for 2 h. The digested peptides were labeled with TMT reagents by dissolving 40 μg of TMT in 2 μL anhydrous ACN, incubating with 50 μL peptide solution at room temperature for 1 hour, and quenching with 1 μL of 5% hydroxylamine for 15 min.

For the In-solution-TMT workflow, 20 μg of mouse brain proteins (8 M urea/HEPES) were reduced with 1.1 μL of 200 mM DTT (10 mM, 37 °C, 60 min), alkylated with 1.12 μL of 800 mM IAA (40 mM, dark, 30 min), and digested with 2 μL trypsin (0.5 μg, 1:40) overnight at 37 °C. Digestion was stopped by adding 2 μL of 1% TFA (final concentration 0.1%). Peptides were desalted with 0.75 mg C18 tips using the following steps: activation with 50 μL 50% ACN/0.1% FA, equilibration with 50 μL 0.1% FA, sample loading in 30 μL 0.1% FA, washing with 50 μL 0.1% FA (×2), and elution with 30 μL 50% ACN/0.1% FA (×2). The eluates were vacuum dried and TMTpro 18-plex (Thermo Scientific) labeling was performed according to the manufacturer's instructions (2 μL TMT reagent in 2 μL anhydrous

ACN, 50 μL peptide solution, 1 hour incubation at room temperature, and quenching with 1 μL of 5% hydroxylamine for 15 min).

The labeling solution were combined, dried, and resolubilized in 200 μL of 80% ACN/6% TFA for phosphopeptide enrichment. IMAC tips containing 1 mg beads were equilibrated with 100 μL of 80% ACN/6% TFA, loaded with 100 μL of sample solution, and sequentially washed with 50 μL 80% ACN/6% TFA, 2 × 100 μL 50% ACN/6% TFA/200 mM NaCl, 2 × 100 μL 30% ACN/0.1% FA, and 2 × 50 μL 30% ACN. Bound phosphopeptides were eluted twice with 30 μL of 10% ammonia, vacuum dried, and stored at -80 °C.

Finally, phosphopeptide eluates, as well as protein fractions from the combined flow-throughs, were dried, resolubilized in 30 μL of 0.1% FA, desalted with 0.75 mg C18 tips (activation: 50 μL 50% ACN/0.1% FA; equilibration: 50 μL 0.1% FA; loading: 30 μL 0.1% FA; wash: 2 × 50 μL 0.1% FA; elution: 2 × 30 μL 50% ACN/0.1% FA), dried again, and stored at -80 °C until LC-MS/MS analysis.

TMTpro18-labeled MS data were processed using MaxQuant (v2.7.5.0) with Andromeda. Reporter ion MS2 quantification was enabled with the TMTpro 18plex channel set. Spectra were searched against the UniProt mouse reference proteome using trypsin/P specificity, fixed carbamidomethylation (C) and TMTpro18 (K/N-term), and variable oxidation (M), acetylation (protein N-term), and phospho (STY) for phospho-enriched samples. MS1/MS2 tolerances were 4.5/20 ppm, and identifications were filtered at 1% FDR. Reporter intensities were interference-corrected and exported for downstream quantitative analysis.

Integrated proteome, glycoproteome, and phosphoproteome analysis of aging mouse samples and NB4 cells using the MuPPE platform: For each aging mouse sample (choroid plexus, CSF, and serum) and for NB4 cell lysates, total protein concentration was quantified using the BCA Protein Assay Kit (Beyotime, China) following the manufacturer's instructions. Exactly 50 μg of total protein from each sample was used as input for the subsequent MuPPE workflow.

In MuPPE process: Denaturation, PAC and on-line digestion on Click-Maltose. Then, adjust the tryptic sample solution to 80% ACN/1% TFA by adding ACN and TFA and incubate for 10 min for glycopeptides enrichment. Finally, adjust the flow-through condition for phosphopeptide enrichment according to the manufacturer's instructions, and then incubate for 10 min to facilitate phosphopeptide enrichment. The remaining (flow-through) peptides not bound in the glyco/phosphopeptides enrichment

steps were retained for proteomics. All peptide fractions were analyzed using LC-MS/MS platforms. For data processing and integration across the three layers, contaminants and decoys were removed. PTM site localization used engine-specific criteria: for the phosphoproteome we retained Class-I sites with Localization Probability>0.75 and Site q-value <0.01 (peptide/precursor q<0.01); results were robust at a stricter 0.90 cutoff. For the glycoproteome we controlled 2D-FDR=1% at the glyco-PSM level and required Byonic Score>150; Raw intensities were run-level TIC-normalized by scaling each run's total precursor signal to the cohort median (scale factor $\alpha_r = \mathrm{median(TIC)/TIC}_r$) and then $\log_2$-transformed. To ensure both depth and reliability, layer-specific coverage thresholds were applied: features in the proteome and phosphoproteome were retained only if quantified in >80% of replicates within at least one group, whereas the glycoproteome retained features quantified in ≥50% of replicates, acknowledging the higher intrinsic sparsity of glycopeptide data. Missing values were then handled in a layer-specific manner: for the proteome and phosphoproteome, partially missing entries were imputed within layer on log2-transformed data using the k-nearest neighbors algorithm (KNN, k = 5), while for the glycoproteome, missing values were treated as true non-detections and replaced with 0 at the raw scale (followed by ε + log2 transformation to avoid undefined values). Finally, for cross-layer comparability, all features were Z-scored within each layer (per feature across samples) before integration and downstream statistical analyses.

Benchmark workflow

MuPPE workflow: Mouse brain lysates (20 μg) were diluted to 1 μg/μL and incubated with 0.6 mg maltose beads for 10 min, followed by addition of 63 μL ACN. Samples were washed three times with 200 μL of 80% ethanol, then resolubilized in 8 μL of 6 M urea and diluted with 40 μL of ammonium bicarbonate. Proteins were digested with 0.5 μg trypsin for 2.5 h, and the reaction was quenched with 2.6 μL TFA and 206.3 μL ACN. After 15 min incubation, glycopeptides were captured by centrifugation. Maltose beads were washed three times with 50 μL of 80% ACN/1% TFA and eluted with 30 μL of 30% ACN/1% FA (×3). Flow-through and wash fractions were combined, vacuum-dried, and incubated with 0.5 mg IMAC beads in 50 μL 80% ACN/6% TFA for 1 hour. IMAC beads were sequentially washed with 50 μL 80% ACN/6% TFA, 2 × 100 μL 50% ACN/6% TFA/200 mM NaCl, 2 × 200 μL 30% ACN/0.1% FA, and 2 × 100 μL 30% ACN. Phosphopeptides were finally eluted twice with 30 μL 10% ammonia solution and pooled.

In-solution digestion: Mouse brain lysates (20 μg) were denatured in 8 M urea with protease inhibitor cocktail (final 1 μg/μL). Proteins were reduced with 1 μL 200 mM DTT and alkylated with 1 μL 400 mM IAA. After dilution with 140 μL ammonium bicarbonate, proteins were digested with 0.5 μg trypsin. Digestion was quenched, and peptides were desalted using C18 SPE columns: columns were activated, samples loaded, washed with 20 μL water/0.1% FA, and eluted with 2 × 20 μL 50% ACN/0.1% FA. Eluates were vacuum-dried. For glycopeptide enrichment, 20 μg desalted peptides were dissolved in 30 μL 80% ACN/1% TFA and incubated with 0.6 mg maltose beads for 15 min. Beads were washed (80% ACN/1% TFA, 50 μL × 3) and eluted (30% ACN/1% FA, 30 μL × 3). For phosphopeptide enrichment, 20 μg peptides were dissolved in 50 μL 80% ACN/6% TFA and incubated with 0.5 mg IMAC beads for 1 hour. After washing as described above, phosphopeptides were eluted twice with 30 μL 10% ammonia solution and pooled.

Reference methods: For benchmarking, two reported workflows were included. In method 1[10], 20 μg digested peptides were dissolved in 50 μL 80% ACN/1% TFA, loaded onto a maltose-packed tip column, and processed as described above. The flow-through was dried, resolubilized in 50 μL 80% ACN/6% TFA, and incubated with 0.5 mg IMAC beads, followed by washing and elution as above. In method 2[11], 20 μg desalted peptides were dissolved in 50 μL 80% ACN/6% TFA and incubated with 0.5 mg IMAC beads for 1 hour. Beads were washed as above, and phosphopeptides were eluted with 30 μL 10% ammonia (twice). The IMAC flow-through was concentrated, loaded onto OASIS MAX columns (1 mg), equilibrated with 95% ACN/1% TFA, and subjected to sequential washing and elution according to the original protocol.

Normalization of PTMs abundance to protein level: To account for potential changes in total protein expression and to accurately assess site-specific regulation, we normalized the abundance of each PTMs site (phosphorylation or glycosylation) to the corresponding parent protein abundance in each sample. Specifically, for every quantified PTMs site and sample, we calculated the normalized value as the $\log_2$-transformed ratio of the PTMs site intensity to the parent protein intensity:

$$\text{Normalized PTM abundance} = \log_2(\text{PTM site intensity}) - \log_2(\text{protein intensity});$$

We assessed potential protein-level confounding by pairing each quantified PTMs site with its parent protein and plotting site $\log_2$ fold-change versus protein $\log_2$ fold-change for each contrast. As a rule,

when PTMs regulation was not coupled to protein abundance (i.e., regulated sites with parent proteins showing no detectable change), we performed downstream analyses on site-level intensities after standard sample-level normalization. Because dividing by protein restricts coverage to sites with complete protein co-quantification, protein-division was not used for the primary results; matched protein values are provided in Supplementary Data.

Flow cytometry analysis: Cellular DNA was stained with propidium iodide (PI) to assess cell cycle distribution and apoptosis. Briefly, harvested cells were washed with phosphate-buffered saline (PBS) and fixed in pre-chilled 70% ethanol at 4°C for 12 hours. After removing the ethanol, the cells were resuspended in a staining buffer containing PI and RNase A (0.5 mL per tube) and then were incubated at 37°C in the dark for 30 min. PI fluorescence was detected in the B690-PC5.5 channel of a flow cytometer (excitation wavelength, 488 nm), while forward and side scatter characteristics were simultaneously analyzed. Cell cycle phases and apoptotic cells were determined based on DNA content distribution, where the G0/G1 peak set to 1, the G2/M peak as approximately 2, and the sub-G1 peak was identified as indicative of apoptosis. Detection was performed using a flow cytometer (Beckman Coulter CytoFLEX S).

Supplementary References

1. Chen, Z. et al. In-depth Site-specific Analysis of N-glycoproteome in Human Cerebrospinal Fluid and Glycosylation Landscape Changes in Alzheimer's Disease. *Mol. Cell. Proteomics* **20**, 100081 (2021).
2. Guldbrandsen, Astrid et al. In-depth Characterization of the Cerebrospinal Fluid (CSF) Proteome Displayed Through the CSF Proteome Resource (CSF-PR). *Mol. Cell. Proteomics* **13**, 3152-3163 (2014).
3. Wang, D. et al. Boost-DiLeu: Enhanced Isobaric N, N-Dimethyl Leucine Tagging Strategy for a Comprehensive Quantitative Glycoproteomic Analysis. *Anal. Chem*. **94**, 11773-11782 (2022).
4. Liu, L. & Duff, K. A technique for serial collection of cerebrospinal fluid from the cisterna magna in mouse. *J. Vis. Exp.* **10**, 960 (2008).
5. Yu, L. et al. Hydrophilic interaction chromatography-based enrichment of glycopeptides by using click maltose: a matrix with high selectivity and glycosylation heterogeneity coverage. *Chem. Eur. J.* **15**, 12618-12626 (2009).
6. Shen, A. et al. Preparation and chromatographic evaluation of a cysteine-bonded zwitterionic hydrophilic interaction liquid chromatography stationary phase. *J. Chromatogr. A* **1228,** 175-182 (2012).
7. Dong, X. et al. In-depth analysis of glycoprotein aialylation in serum using a dual-functional material with superior hydrophilicity and switchable surface charge. *Anal. Chem*. **89**, 3966-3972 (2017).
8. Huang, J. et al. Development of a computational tool for automated interpretation of intact *O*-glycopeptide tandem mass spectra from single proteins. *Anal. Chem.* **92**, 6777-6784 (2020).
9. Cao, L. et al. Application of a strong anion exchange material in electrostatic repulsion-hydrophilic interaction chromatography for selective enrichment of glycopeptides. *J. Chromatogr. A* **1299**, 18-24 (2013).
10. Ding, X. et al. Tandem HILIC-IMAC strategy for simultaneous N-glycoproteomics and phosphoproteomics in aging mouse brain. *J. Chromatogr. A* **1739** (2025).
11. Zhou, Y. et al. An integrated workflow for global, glyco-, and phospho-proteomic analysis of tumor tissues. *Anal. Chem.* **92**, 1842-1849 (2019).
