# Supplementary material for: A versatile platform for sequential glyco-, phospho-, and proteomics with multi-PTMs integration
Source: Nat Commun. 2026 Jan 28;17:948. doi: 10.1038/s41467-025-68270-7 (PMC12852849; doi:10.1038/s41467-025-68270-7)
Supplement: Supplementary file 2 — Reporting Summary [file 41467_2025_68270_MOESM2_ESM.pdf]

nature portfolio

Corresponding author(s): Xiuling Li

Last updated by author(s): Dec 8, 2025

Reporting Summary

Nature Portfolio wishes to improve the reproducibility of the work that we publish. This form provides structure for consistency and transparency in reporting. For further information on Nature Portfolio policies, see our Editorial Policies and the Editorial Policy Checklist.

Statistics

For all statistical analyses, confirm that the following items are present in the figure legend, table legend, main text, or Methods section.

n/a	Confirmed	
☐	☒	The exact sample size (*n*) for each experimental group/condition, given as a discrete number and unit of measurement
☐	☒	A statement on whether measurements were taken from distinct samples or whether the same sample was measured repeatedly
☐	☒	The statistical test(s) used AND whether they are one- or two-sided<br>*Only common tests should be described solely by name; describe more complex techniques in the Methods section.*
☒	☐	A description of all covariates tested
☐	☒	A description of any assumptions or corrections, such as tests of normality and adjustment for multiple comparisons
☒	☐	A full description of the statistical parameters including central tendency (e.g. means) or other basic estimates (e.g. regression coefficient) AND variation (e.g. standard deviation) or associated estimates of uncertainty (e.g. confidence intervals)
☐	☒	For null hypothesis testing, the test statistic (e.g. *F*, *t*, *r*) with confidence intervals, effect sizes, degrees of freedom and *P* value noted<br>*Give P values as exact values whenever suitable.*
☒	☐	For Bayesian analysis, information on the choice of priors and Markov chain Monte Carlo settings
☒	☐	For hierarchical and complex designs, identification of the appropriate level for tests and full reporting of outcomes
☐	☒	Estimates of effect sizes (e.g. Cohen's *d*, Pearson's *r*), indicating how they were calculated

Our web collection on statistics for biologists contains articles on many of the points above.

Software and code

Policy information about availability of computer code

Data collection	Orbitrap 480 (Thermo Fisher Scientific)
Data analysis	Commercial softwares licensed were utilized: for the quantitative analysis of phosphoproteins and glycoproteins, we utilized Spectronaut (v19.0.240606.62635) and Protein Metrics (v5.4.10). Data visualization was performed using GraphPad Prism (version 9.5.1). Weighted Gene Co-expression Network Analysis (WGCNA) was conducted with R (version 4.4.2). Figures were assembled and refined using Adobe Illustrator CC (version 23.0.2). Cytoscape (version 3.10.2) was utilized to construct and visualize glycan co-expression networks, providing a comprehensive view of the interrelationships.

For manuscripts utilizing custom algorithms or software that are central to the research but not yet described in published literature, software must be made available to editors and reviewers. We strongly encourage code deposition in a community repository (e.g. GitHub). See the Nature Portfolio guidelines for submitting code & software for further information.

Data

Policy information about availability of data

All manuscripts must include a data availability statement. This statement should provide the following information, where applicable:

- Accession codes, unique identifiers, or web links for publicly available datasets
- A description of any restrictions on data availability
- For clinical datasets or third party data, please ensure that the statement adheres to our policy

Source data are provided with this paper. All data supporting the findings of this study are available in the Supplementary Information and from the figshare data repository https://figshare.com/s/5f422ef846184ea9df83. The mass spectrometry proteomics data generated in this study have been deposited to the ProteomeXchange Consortium via the iProX partner repository74,75 with the dataset identifier PXD059701 and IPX0009561000. Unless otherwise stated, all data supporting the results of this study can be found in the article, Supplementary and source data files.

Research involving human participants, their data, or biological material

Policy information about studies with human participants or human data. See also policy information about sex, gender (identity/presentation), and sexual orientation and race, ethnicity and racism.

Reporting on sex and gender	This study did not specifically consider sex and gender in its design as [reason, e.g., the study focuses on molecular or cellular mechanisms that are not known to differ by sex]. Participants' sex and gender information was not collected, as it was not deemed relevant to the study objectives. Future studies may explore potential sex or gender differences if they emerge as relevant factors.
Reporting on race, ethnicity, or other socially relevant groupings	The serum and cerebrospinal fluid samples used for methodological development were obtained from participants of Asian ethnicity. These samples were provided by the Second Affiliated Hospital of Dalian Medical University. The classification of ethnicity was based on self-reported demographic information. Confounding variables, such as age or clinical background, were not relevant for this methodological study.
Population characteristics	The population consisted of serum and cerebrospinal fluid samples obtained from human participants of Asian ethnicity. These samples were used exclusively for methodological development. No additional covariates, such as age, genotype, or diagnostic categories, were considered in this study.
Recruitment	Samples were collected from human participants recruited through the Second Affiliated Hospital of Dalian Medical University. Participant selection followed the hospital's standard clinical protocols, and no self-selection bias was present. All participants provided informed consent prior to sample collection.
Ethics oversight	This study was approved by the Ethics Committee of Gan Jiang Chinese Medicine Innovation Center, under the approval number GJCMIC2025-008.

Note that full information on the approval of the study protocol must also be provided in the manuscript.

Field-specific reporting

Please select the one below that is the best fit for your research. If you are not sure, read the appropriate sections before making your selection.

☒ Life sciences ☐ Behavioural & social sciences ☐ Ecological, evolutionary & environmental sciences

For a reference copy of the document with all sections, see nature.com/documents/nr-reporting-summary-flat.pdf

Life sciences study design

All studies must disclose on these points even when the disclosure is negative.

Sample size	For the aging mechanism study, each age group consisted of 14 female and 14 male mice (2 months, 12 months, and 24 months).
Data exclusions	No data were excluded from the analyses. All collected data met pre-defined quality criteria and were included in the study.
Replication	Due to the limited sample size in the mouse aging cohort, samples from each month were pooled by sex into male and female groups. All experiments on the pooled samples were conducted in triplicate to ensure reproducibility.
Randomization	Mice were randomly assigned to experimental age groups. For methodological optimization experiments, randomization was not applicable as the study focused on standardizing protocols rather than testing group-specific outcomes.
Blinding	Blinding was not applicable for this study, as the investigators were aware of the experimental design (e.g., age and sex of the mice) to ensure proper protocol execution. However, for data analysis, automated and unbiased methods were used to minimize potential bias.

Reporting for specific materials, systems and methods

We require information from authors about some types of materials, experimental systems and methods used in many studies. Here, indicate whether each material, system or method listed is relevant to your study. If you are not sure if a list item applies to your research, read the appropriate section before selecting a response.

Materials & experimental systems

n/a	Involved in the study	
☒	☐	Antibodies
☐	☒	Eukaryotic cell lines
☒	☐	Palaeontology and archaeology
☐	☒	Animals and other organisms
☒	☐	Clinical data
☒	☐	Dual use research of concern
☒	☐	Plants

Methods

n/a	Involved in the study	
☒	☐	ChIP-seq
☐	☒	Flow cytometry
☒	☐	MRI-based neuroimaging

Eukaryotic cell lines

Policy information about cell lines and Sex and Gender in Research

Cell line source(s)	The NB4 cell line, derived from human acute promyelocytic leukemia cells, was obtained from [supplier/source, e.g., ATCC or another cell repository]. The cells were maintained in RPMI-1640 medium supplemented with 10% fetal bovine serum at 37° C in a 5% CO_2 atmosphere.
Authentication	The NB4 cell line was authenticated using short tandem repeat (STR) profiling to confirm its identity. Authentication was performed prior to the start of the experiments to ensure validity.
Mycoplasma contamination	The NB4 cell line was tested for mycoplasma contamination using [specific assay, e.g., PCR-based assay or MycoAlert mycoplasma detection kit], and all tests confirmed the absence of contamination.
Commonly misidentified lines (See ICLAC register)	The NB4 cell line is not listed among commonly misidentified cell lines in the ICLAC register and is widely recognized as a valid model for studying acute promyelocytic leukemia.

Animals and other research organisms

Policy information about studies involving animals; ARRIVE guidelines recommended for reporting animal research, and Sex and Gender in Research

Laboratory animals	This study involved laboratory animals, specifically C57BL/6 mice, which were housed under controlled conditions (22 ± 2°C, 50 ± 5% humidity, 12-hour light/dark cycle) with ad libitum access to food and water. The mice were categorized into three age groups: 2 months, 12 months, and 24 months. Each group included 14 male and 14 female mice.
Wild animals	This study did not involve wild animals.
Reporting on sex	Both sexes were considered in the study design. Each age group included an equal number of male (n=14) and female (n=14) mice to account for potential sex-based differences. The sex of the mice was determined based on biological attributes, and all analyses were conducted with disaggregated data for sex.
Field-collected samples	This study did not involve field-collected samples.
Ethics oversight	All animal experiments were approved by the Ethics Committee of Center for Excellence in Molecular Cell Science (approval number: 2025-001) and conducted in compliance with institutional and national guidelines for animal care and use.

Note that full information on the approval of the study protocol must also be provided in the manuscript.

Plants

Seed stocks

Report on the source of all seed stocks or other plant material used. If applicable, state the seed stock centre and catalogue number. If plant specimens were collected from the field, describe the collection location, date and sampling procedures.

Novel plant genotypes

Describe the methods by which all novel plant genotypes were produced. This includes those generated by transgenic approaches, gene editing, chemical/radiation-based mutagenesis and hybridization. For transgenic lines, describe the transformation method, the number of independent lines analyzed and the generation upon which experiments were performed. For gene-edited lines, describe the editor used, the endogenous sequence targeted for editing, the targeting guide RNA sequence (if applicable) and how the editor was applied.

Authentication

Describe any authentication procedures for each seed stock used or novel genotype generated. Describe any experiments used to assess the effect of a mutation and, where applicable, how potential secondary effects (e.g. second site T-DNA insertions, mosiacism, off-target gene editing) were examined.

Flow Cytometry

Plots

Confirm that:

☐ The axis labels state the marker and fluorochrome used (e.g. CD4-FITC).

☐ The axis scales are clearly visible. Include numbers along axes only for bottom left plot of group (a 'group' is an analysis of identical markers).

☐ All plots are contour plots with outliers or pseudocolor plots.

☐ A numerical value for number of cells or percentage (with statistics) is provided.

Methodology

Sample preparation

Describe the sample preparation, detailing the biological source of the cells and any tissue processing steps used.

Instrument

Identify the instrument used for data collection, specifying make and model number.

Software

Describe the software used to collect and analyze the flow cytometry data. For custom code that has been deposited into a community repository, provide accession details.

Cell population abundance

Describe the abundance of the relevant cell populations within post-sort fractions, providing details on the purity of the samples and how it was determined.

Gating strategy

Describe the gating strategy used for all relevant experiments, specifying the preliminary FSC/SSC gates of the starting cell population, indicating where boundaries between "positive" and "negative" staining cell populations are defined.

☐ Tick this box to confirm that a figure exemplifying the gating strategy is provided in the Supplementary Information.
